# Pseudoatrial Flutter: When the Problem Lies Outside the Heart

**DOI:** 10.5811/cpcem.2019.11.44665

**Published:** 2020-01-23

**Authors:** Samuele Ceruti, Marco Spagnoletti, Romano Mauri

**Affiliations:** * Hôpitaux Universitaires de Genève, Department of Intensive Care, Genève, Switzerland; † Clinica Luganese, Via Moncucco, Department of Emergency Medicine, Lugano, Switzerland

## Abstract

Electrocardiogram (ECG) artifacts are a common problem in emergency medicine. Generally these artifacts are induced by movement disorders, which generate electrical interference with the ECG recording. If these disorders are not promptly recognized, consequences can lead to hospitalization and execution of unnecessary diagnostic tests, thereby increasing the costs and clinical risks such as nosocomial infections and thromboembolism. We present a pseudoatrial flutter generated by a Parkinson’s-like movement.

## CASE PRESENTATION

A 72-year-old woman presented to the emergency department with chest pain for several days, not associated with dyspnea or other symptoms. She reported that similar symptoms had occurred in the past. The patient was quickly assessed with the performance of an electrocardiogram (ECG), which demonstrated the following tracing (Image). After a first suspicion of a paroxysmal atrial flutter, it was noted that precordial “pseudo F” waves were really broad in amplitude, while a sinus rhythm was maintained in lead three. Moreover, even with the disappearance of the “pseudo F” waves, it was also noted that the QRS complexes remained regular between the suspected “flutter phase” and the “sinus phase,” without any compensatory pauses or changes in heart rate. An artifact ECG was likely: the lack of “pseudo F” waves in lead three increased the probability of artifact involving the right peripheral electrode, thus saving lead three, which analyzes only the electrode of the left foot.

A new ECG recording was performed by the physician verifying electrode placement, and it revealed the appearance of artifacts quite similar to the previous one. Again, pseudo-F waves were noted in all leads except for lead three (not shown), although this signal was larger and not completely identical to the previous one recorded. At the end of this recording, the clinicians noted a slight rhythmic tremor resembling Parkinsonism at 5–6 hertz in the right arm, which was thought to be responsible for the recorded electrical signal (Video).

## DISCUSSION

ECG artifacts are common in emergency situations, especially among patients with movement disorders such as Parkinsonism,^1^,^2^ simulating some arrhythmias such as atrial flutter.^3^,^4^ The role of a clinician is to identify these anomalies and promptly look for extra-cardiac conditions,^5^–^6^ to avoid any inappropriate and potentially dangerous consequences such as hospitalization, risk of infection, use of unnecessary diagnostic tests and procedures, and increased patient anxiety.^6^ The patient’s symptoms were ultimately found to be non-cardiac in nature and she was subsequently discharged home in improved clinical status.

**CPC-EM Capsule**

What do we already know about this clinical entity?

Electrocardiogram (ECG) artifacts are common in emergency situations, especially among patients with movement disorders.

What is the major impact of the image(s)?

In analyzing the ECG, it is possible to trace the lead affected by the artifact and identify a movement disorder.

How might this improve emergency medicine practice?

The clinician’s role is to identify these anomalies in order to avoid dangerous consequences such as hospitalization and unnecessary diagnostic procedures.

## Supplementary Information

Video. Patient’s right arm Parkinsonism, noted just after electrocardiographic recording, was responsible for the artifact electrocardiogram.

## Figures and Tables

**Image:**
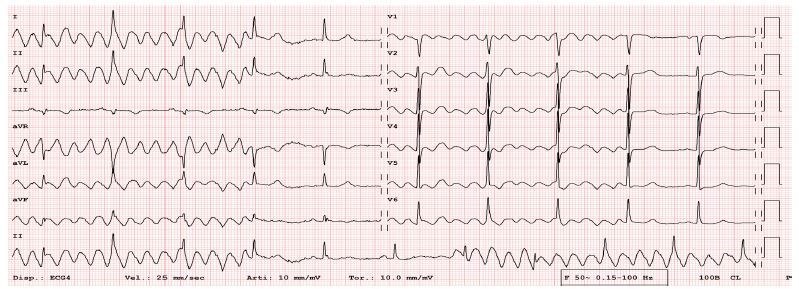
Patient’s artifact electrocardiogram showing pseudoatrial flutter. Note regular maintenance of QRS complexes between the suspected “flutter phase” (arrowheads) and the “sinus phase”; moreover, lead three demonstrated continuous sinus rhythm (arrows).
